# Supplementary material for: Epiallelic variation of non-coding RNA genes and their phenotypic consequences
Source: Nat Commun. 2024 Feb 14;15:1375. doi: 10.1038/s41467-024-45771-5 (PMC10867003; doi:10.1038/s41467-024-45771-5)
Supplement: Supplementary file 1 — Supplementary Information [file 41467_2024_45771_MOESM1_ESM.pdf]

Supplementary Figures for: Epiallelic variation of non-coding RNA genes and their phenotypic consequences

Jie Liu[1], Xuehua Zhong[1*]

[1] Department of Biology, Washington University in St. Louis, St. Louis, MO 63130, USA

[*] Author for correspondence:
Xuehua Zhong: *xuehuazhong@wustl.edu*

Supplementary Fig. 1 | GWAS of CHH methylation of CMT2 targeted TEs.
Supplementary Fig. 2 | GWAS of CG methylation of AT4G05535.
Supplementary Fig. 3 | GWAS of CHG methylation of AT2G06395.
Supplementary Fig. 4 | GWAS of CG, CHH methylation of miR157a.
Supplementary Fig. 5 | Flowering time of pMIR157A:MIR157A.
Supplementary Fig. 6 | Pearson's correlation between abundance of miR157a and flowering time.
Supplementary Fig. 7 | CHG and CHH DNA methylation of miR157a in WT and SunTag-MQ1.
Supplementary Fig. 8 | RT-qPCR results of *SPL* genes in WT and SunTag-MQ1 line.
Supplementary Fig. 9 | LD decay calculated with genotype data of 811 accessions.
Supplementary Fig. 10 | Density and cumulative proportion plot of significant SNP number in 16Kb region and QTL number.

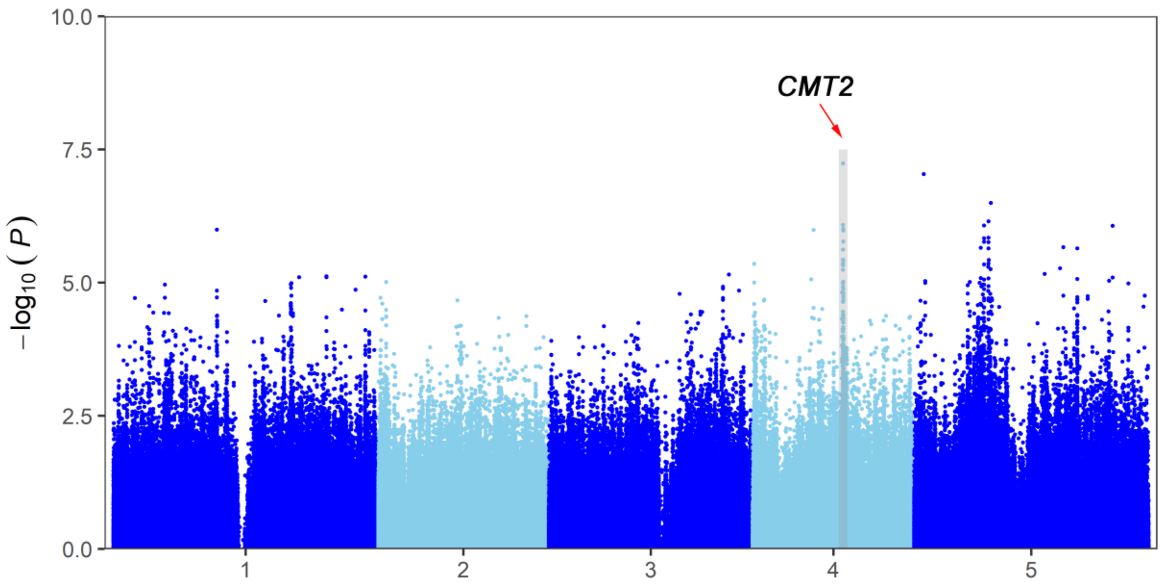

Supplementary Fig. 1 | GWAS of CHH methylation of CMT2 targeted TEs.
Manhattan plot of GWAS of CHH methylation of CMT2 targeted TEs. Each dot indicated each SNP used in GWAS. The gray box showed the significant signal around *CMT2*.

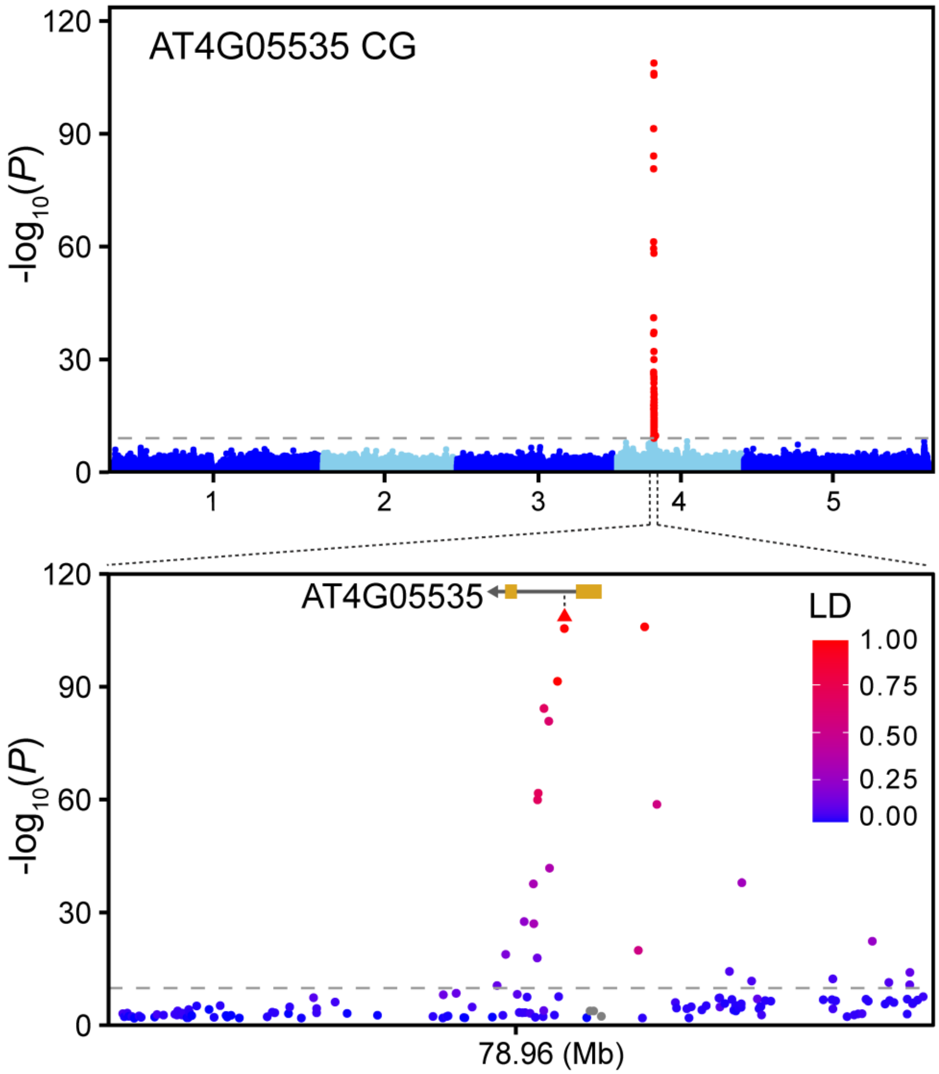

Supplementary Fig. 2 | GWAS of CG methylation of AT4G05535.

Manhattan plot of GWAS of CG methylation of AT4G05535 (top panel). The red dots indicated significant SNPs. The bottom panel showed the zoom-in view of the significant region on Chr4. The red triangle showed the most significant SNP located in the intron region of AT4G05525. The colors of the dots indicated the LD between each SNP with the most significant SNP.

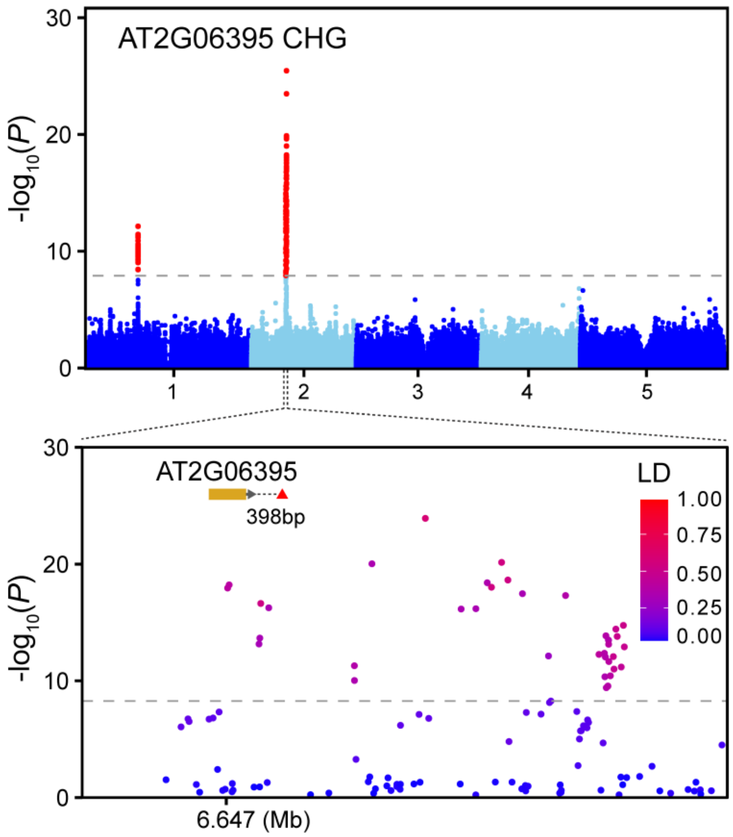

Supplementary Fig. 3 | GWAS of CHG methylation of AT2G06395.

Manhattan plot of GWAS result of CHG methylation of AT2G06395 (top panel). The red dots indicated significant SNPs. The bottom panel showed the zoom-in view of the significant region on Chr2. The red triangle showed the most significant SNP located in the downstream region of AT2G06395. The colors of the dots indicated the LD between each SNP with the most significant SNP.

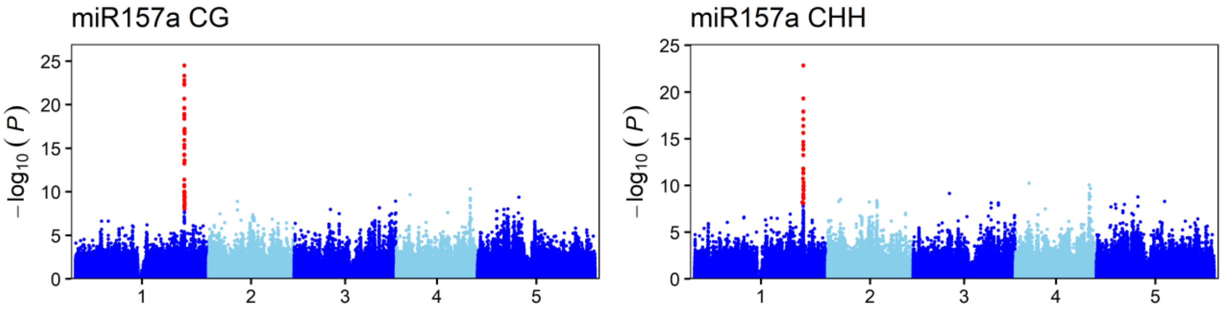

Supplementary Fig. 4 | GWAS of CG, CHH methylation of miR157a.
Manhattan plot of GWAS of CG (left) and CHH (right) methylation of miR157a. The red dots indicated significant SNPs.

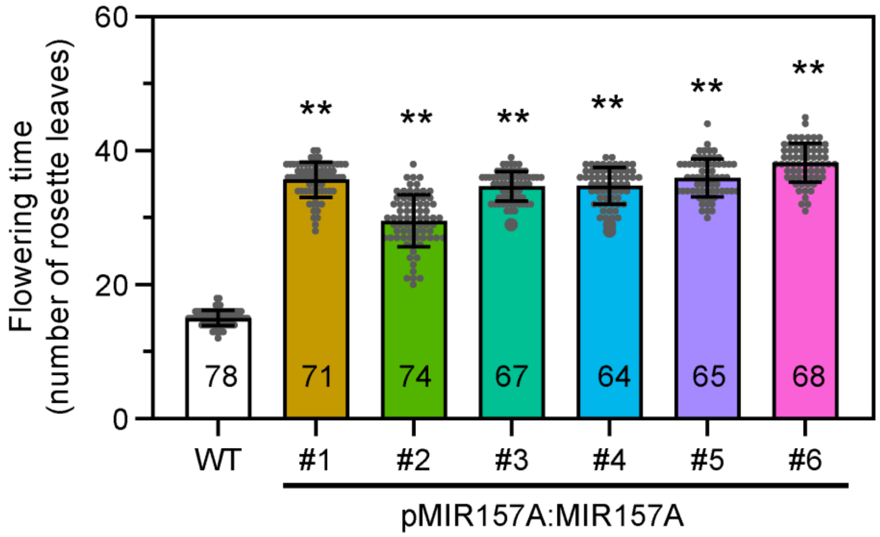

Supplementary Fig. 5 | Flowering time of pMIR157A:MIR157A.

Flowering phenotype of WT and *pMIR157A:MIR157A* transgenic lines. The number in each bar is the number of plants used for phenotyping. The *P*-value is calculated by one-way ANOVA analysis. Data are presented as mean ± SD. **, $P < 0.01$.

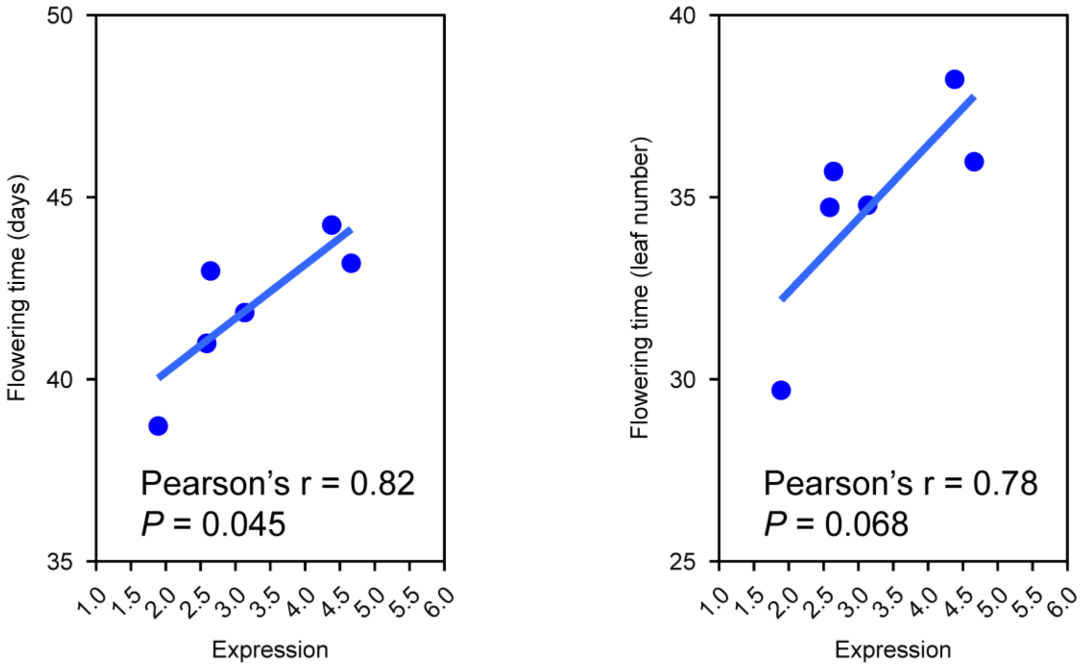

Supplementary Fig. 6 | Pearson's correlation between abundance of miR157a and flowering time. The expression level and flowering time were measured in pMIR157A:MIR157A transgenic lines. The correlation analysis (Pearson's r and *P*-value) was performed with "corr.test" in R "psych" package.

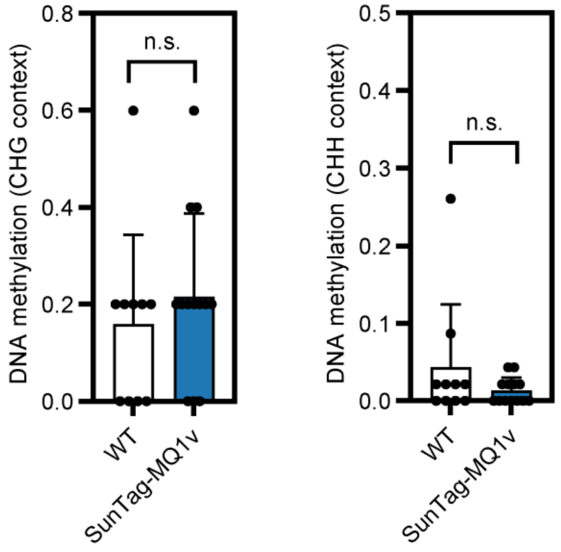

Supplementary Fig. 7 | CHG and CHH DNA methylation of miR157a in WT and SunTag-MQ1 transgenic line.

There were no significant differences of CHG (left) and CHH (right) DNA methylation of miR157a in WT and SunTag-MQ1. The one-way ANOVA was used to test the significance of difference. No significant difference was observed. N = 10 and 13 for WT and SunTag-MQ1, respectively. Data are presented as mean ± SD.

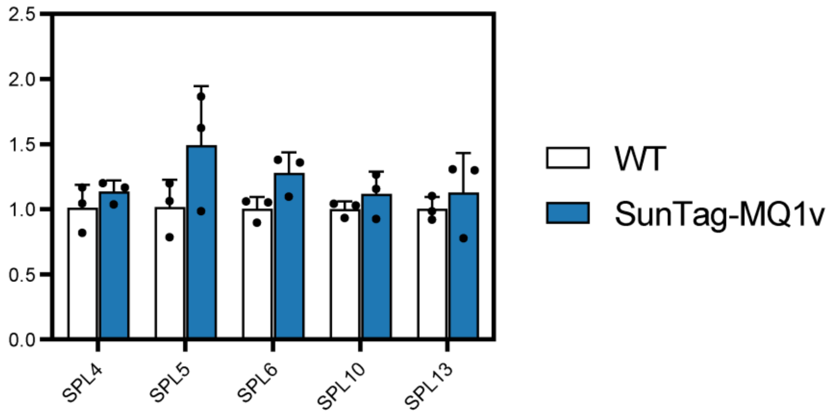

Supplementary Fig. 8 | RT-qPCR results of *SPL* genes in WT and SunTag-MQ1 transgenic line. Expression levels of *SPL* genes measured with RT-qPCR. For each measurement, three replicates are used. The *P*-value is calculated by one-way ANOVA analysis. No significant difference was observed. Data are presented as mean ± SD.

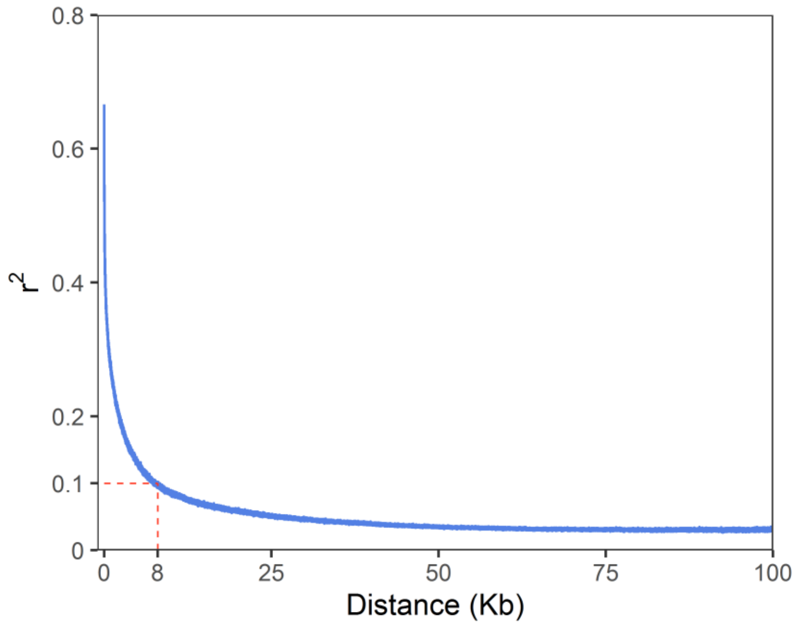

Supplementary Fig. 9 | LD decay calculated with genotype data of 811 accessions.

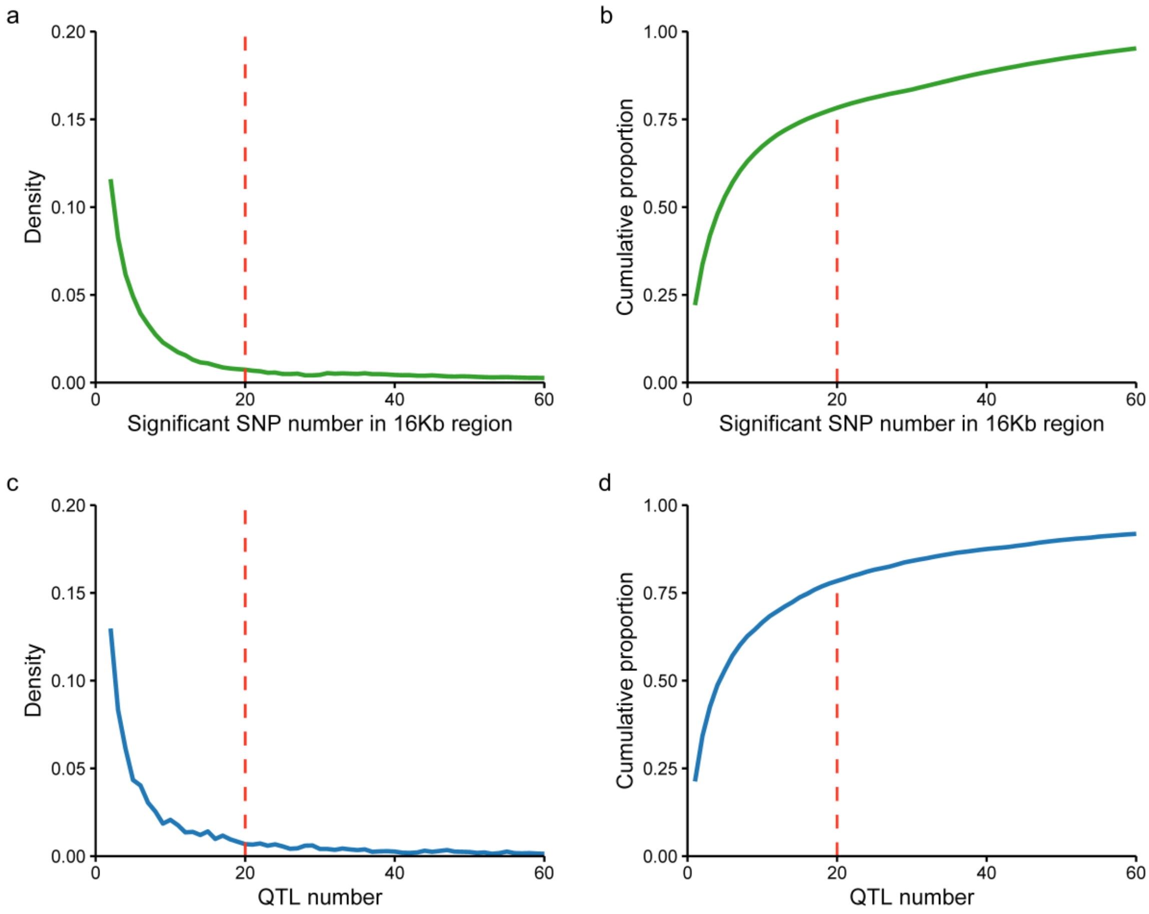

Supplementary Fig. 10 | Density and cumulative proportion plot of significant SNP number in 16Kb region and QTL number.
