# Supplementary material for: Epiallelic variation of non-coding RNA genes and their phenotypic consequences
Source: Nat Commun. 2024 Feb 14;15:1375. doi: 10.1038/s41467-024-45771-5 (PMC10867003; doi:10.1038/s41467-024-45771-5)
Supplement: Supplementary file 3 — Description of Additional Supplementary Files [file 41467_2024_45771_MOESM3_ESM.pdf]

Description of Additional Supplementary Files

File Name: Supplementary Data 1
Description: 811 Arabidopsis accessions used in GWAS analysis

File Name: Supplementary Data 2
Description: Statistic of DNA methylation of ncRNA genes in 811 accessions

File Name: Supplementary Data 3
Description: Significant correlations between DNA methylation of ncRNA genes and latitude/longitude of natural accessions

File Name: Supplementary Data 4
Description: Significant correlation between DNA methylation of ncRNA genes and 303 phenotypes of natural accessions

File Name: Supplementary Data 5
Description: Detail information of 496 methylQTL

File Name: Supplementary Data 6
Description: The overlap of significant SNPs and genomic features

File Name: Supplementary Data 7
Description: Primers used in this study
