# Supplementary material for: Epiallelic variation of non-coding RNA genes and their phenotypic consequences
Source: Nat Commun. 2024 Feb 14;15:1375. doi: 10.1038/s41467-024-45771-5 (PMC10867003; doi:10.1038/s41467-024-45771-5)
Supplement: Supplementary file 11 — Reporting Summary [file 41467_2024_45771_MOESM11_ESM.pdf]

nature portfolio

Corresponding author(s): Xuehua Zhong

Last updated by author(s): Jan 12, 2024

Reporting Summary

Nature Portfolio wishes to improve the reproducibility of the work that we publish. This form provides structure for consistency and transparency in reporting. For further information on Nature Portfolio policies, see our Editorial Policies and the Editorial Policy Checklist.

Statistics

For all statistical analyses, confirm that the following items are present in the figure legend, table legend, main text, or Methods section.

n/a	Confirmed	
☐	☒	The exact sample size (n) for each experimental group/condition, given as a discrete number and unit of measurement
☐	☒	A statement on whether measurements were taken from distinct samples or whether the same sample was measured repeatedly
☐	☒	The statistical test(s) used AND whether they are one- or two-sided<br>*Only common tests should be described solely by name; describe more complex techniques in the Methods section.*
☒	☐	A description of all covariates tested
☐	☒	A description of any assumptions or corrections, such as tests of normality and adjustment for multiple comparisons
☐	☒	A full description of the statistical parameters including central tendency (e.g. means) or other basic estimates (e.g. regression coefficient) AND variation (e.g. standard deviation) or associated estimates of uncertainty (e.g. confidence intervals)
☐	☒	For null hypothesis testing, the test statistic (e.g. F, t, r) with confidence intervals, effect sizes, degrees of freedom and P value noted<br>*Give P values as exact values whenever suitable.*
☒	☐	For Bayesian analysis, information on the choice of priors and Markov chain Monte Carlo settings
☒	☐	For hierarchical and complex designs, identification of the appropriate level for tests and full reporting of outcomes
☐	☒	Estimates of effect sizes (e.g. Cohen's d, Pearson's r), indicating how they were calculated

Our web collection on statistics for biologists contains articles on many of the points above.

Software and code

Policy information about availability of computer code

Data collection	Publicly available Arabidopsis genetic and methylation data were downloaded from NCBI (GEO accession: GSE43857 for methylomes, https://1001genomes.org/data/GMI-MPI/releases/v3.1/1001genomes_snp-short-indel_only_ACGTN.vcf.gz for SNPs) with wget 1.14.
Data analysis	EMMAX was used for GWAS. BEDtools (v2.29.0) was used for methylation calculation. PLINK (v1.9) was used to calculate LD. R software (v3.6.1) was used to perform statistic analysis and plot. Excel (Microsoft 365) was used for one-way ANOVA analysis.

For manuscripts utilizing custom algorithms or software that are central to the research but not yet described in published literature, software must be made available to editors and reviewers. We strongly encourage code deposition in a community repository (e.g. GitHub). See the Nature Portfolio guidelines for submitting code & software for further information.

Data

Policy information about availability of data

All manuscripts must include a data availability statement. This statement should provide the following information, where applicable:

- Accession codes, unique identifiers, or web links for publicly available datasets
- A description of any restrictions on data availability
- For clinical datasets or third party data, please ensure that the statement adheres to our policy

There is no sequencing data or mass spectrometry data generated in this study. The published 1001 Methylomes could be obtained under GEO accession GSE43857

(https://www.ncbi.nlm.nih.gov/geo/query/acc.cgi?acc=GSE43857). All data generated or analyzed during this study are included in this published article. Source data are provided with this paper.

Research involving human participants, their data, or biological material

Policy information about studies with human participants or human data. See also policy information about sex, gender (identity/presentation), and sexual orientation and race, ethnicity and racism.

Reporting on sex and gender	There is no sex- or gender-based analyses. This study only uses plants and there is no sex-related conclusions.
Reporting on race, ethnicity, or other socially relevant groupings	N/A
Population characteristics	N/A
Recruitment	N/A
Ethics oversight	N/A

Note that full information on the approval of the study protocol must also be provided in the manuscript.

Field-specific reporting

Please select the one below that is the best fit for your research. If you are not sure, read the appropriate sections before making your selection.

☒ Life sciences ☐ Behavioural & social sciences ☐ Ecological, evolutionary & environmental sciences

For a reference copy of the document with all sections, see nature.com/documents/nr-reporting-summary-flat.pdf

Life sciences study design

All studies must disclose on these points even when the disclosure is negative.

Sample size	For GWAS analysis, 811 Arabidopsis accessions which is a subset of both Arabidopsis 1001 Genomes and Methylomes project were used. the These 811 accessions have methylomes under same growth temperature. When investigating flowering time, 58-92 plants were used for each genotype. For BS-PCR, 10 and 13 colonies were randomly selected and sequenced. Sample sizes were chosen based on similar sample size generally employed in the field. No statistical approach was used to predetermine sample size.
Data exclusions	During the GWAS analysis, the SNPs with minor allele frequency (MAF) less than 5% were excluded since this method is hard to test associations for rare alleles. During RT-qPCR, one out of three replicates with abnormal Ct value was excluded for SPL10 in OE#2 and SPL5 in KD#2. Outlier values (not in the range of original mean+-SD) of flowering time in each group were excluded.
Replication	For knock-down and over-expression of miR157a, there were two independent transgene lines used. For RT-qPCR, three technical replicates were performed for each biological replicate. All attempts to replicate the experiments in this study were successful.
Randomization	When performing BS-PCR for methylation quantification, colonies for each sample were randomly chose for Sanger sequencing. For all the plant experiments, the samples within each group were taken randomly.
Blinding	Researchers were not blinded to genotypes when we grew and and collect plant tissues. When measuring the plant flowering, we count the days from planting to flowering and the leaf number when plants are flowering. The data collection is objective since we applied same standard to all investigated plants.

Reporting for specific materials, systems and methods

We require information from authors about some types of materials, experimental systems and methods used in many studies. Here, indicate whether each material, system or method listed is relevant to your study. If you are not sure if a list item applies to your research, read the appropriate section before selecting a response.

Materials & experimental systems

n/a	Involved in the study
☒	☐ Antibodies
☒	☐ Eukaryotic cell lines
☒	☐ Palaeontology and archaeology
☒	☐ Animals and other organisms
☒	☐ Clinical data
☒	☐ Dual use research of concern
☐	☒ Plants

Methods

n/a	Involved in the study
☒	☐ ChIP-seq
☒	☐ Flow cytometry
☒	☐ MRI-based neuroimaging

Dual use research of concern

Policy information about dual use research of concern

Hazards

Could the accidental, deliberate or reckless misuse of agents or technologies generated in the work, or the application of information presented in the manuscript, pose a threat to:

No	Yes
☒	☐ Public health
☒	☐ National security
☒	☐ Crops and/or livestock
☒	☐ Ecosystems
☒	☐ Any other significant area

Experiments of concern

Does the work involve any of these experiments of concern:

No	Yes
☒	☐ Demonstrate how to render a vaccine ineffective
☒	☐ Confer resistance to therapeutically useful antibiotics or antiviral agents
☒	☐ Enhance the virulence of a pathogen or render a nonpathogen virulent
☒	☐ Increase transmissibility of a pathogen
☒	☐ Alter the host range of a pathogen
☒	☐ Enable evasion of diagnostic/detection modalities
☒	☐ Enable the weaponization of a biological agent or toxin
☒	☐ Any other potentially harmful combination of experiments and agents

Plants

Seed stocks	All of the seeds used in this study are Arabidopsis Col-0 background, including some transgenic lines. These seeds were collected in greenhouse in the University of Wisconsin-Madison and Washington University in St. Louis.
Novel plant genotypes	The transgenic Arabidopsis plants in this study were generated through the floral dipping method. For knock-down and overexpression, two independent transgenic lines were used. For DNA methylation editing, one transgenic line was used. The experiments were done using homozygous transgenic T3 or T4 lines. For the segregation analysis, F2 population lines from heterozygous transgenic line were used. For dCas9 targeting, the guide sequences used are GTTAATTGTTTGAGAATTGAG and GAGACAATAAAGCAAATAAA.
Authentication	For the transgenic plants, PCR and antibiotics resistance were used to confirm the transgene. For knock-down and overexpression transgenic lines, segregation analysis were performed to make sure that only single insertion event happened. For SunTag lines, two independent F2 population were used to exclude the possible effect of T-DNA insertion.
